# Intra-Articular Formulation of GE11-PLGA Conjugate-Based NPs for Dexamethasone Selective Targeting—In Vitro Evaluation

**DOI:** 10.3390/ijms19082304

**Published:** 2018-08-06

**Authors:** Enrica Chiesa, Silvia Pisani, Barbara Colzani, Rossella Dorati, Bice Conti, Tiziana Modena, Kevin Braeckmans, Ida Genta

**Affiliations:** 1 Department of Drug Sciences, University of Pavia, V.le Taramelli 12, 27100 Pavia (PV), Italy; enrica.chiesa01@universitadipavia.it (E.C.); silvia.pisani01@universitadipavia.it (S.P.); barbara.colzani01@universitadipavia.it (B.C.); rossella.dorati@unipv.it (R.D.); bice.conti@unipv.it (B.C.); tiziana.modena@unipv.it (T.M.); 2 Laboratory of General Biochemistry and Physical Pharmacy, Ghent University, Ottergemsesteenweg 460, 9000 Gent, Belgium; Kevin.Braeckmans@UGent.be

**Keywords:** EGFR targeting, GE11 peptide, PLGA-PEG nanoparticles, chitosan thermosetting hydrogel, intra-articular injection

## Abstract

Selectively targeted nanoscale drug delivery systems have recently emerged as promising intravenously therapeutic option for most chronic joint diseases. Here, a newly synthetized dodecapeptide (GE11)-polylactide-*co*-glycolide (PLGA)-based conjugate was used to prepare *smart* nanoparticles (NPs) intended for intra-articular administration and for selectively targeting Epidermal Growth Factor Receptor (EGFR). GE11-PLGA conjugate-based NPs are specifically uptaken by EGFR-overexpressed fibroblast; such as synoviocytes; which are the primarily cellular component involved in the development of destructive joint inflammation. The selective uptake could help to tune drug effectiveness in joints and to decrease local and systemic side effects. Dexamethasone (DXM) is a glucorticoid drug commonly used in joint disease treatment for both systemic and local administration route. In the present research; DXM was efficiently loaded into GE11-PLGA conjugate-based NPs through an eco-friendly nanoprecipitation method set up for this purpose. DXM loaded GE11-PLGA conjugate-based NPs revealed satisfactory ex vivo cytocompatibility; with proper size (≤150 nm) and good dimensional stability in synovial fluid. Intra-articular formulation was developed embedding DXM loaded GE11-PLGA conjugate-based NPs into thermosetting chitosan-based hydrogel; forming a biocompatible composite hydrogel able to quickly turn from liquid state into gel state at physiological temperature; within 15 min. Moreover; the use of thermosetting chitosan-based hydrogel extends the local release of active agent; DXM.

## 1. Introduction

Corticosteroid intra-articular (IA) injection is a long-established treatment for patients with chronic and non-curable joint diseases, such as rheumatoid arthritis (RA) and most different arthritis-related forms (i.e., osteoarthritis, a chronic RA-like inflammatory arthritis, post-traumatic arthritis or osteoarthritis, and pseudo gout, an acutely painful inflammatory condition) [1,2,3,4].

Although chronic joint diseases differ in etiology and relative risk factors, symptoms, prognoses, and treatments, growing numbers of systemic administered drugs can be employed to manage, alone or in combination, the joint disorders [5]. Non-steroidal anti-inflammatory drugs (NSAIDs), glucocorticoids (GCs), opioids, anti-rheumatic agents (i.e., methotrexate, hydroxychloroquine, sulfasalazine, and clodronate), and more recently biological agents (i.e., adalimumab, infliximab, and etanercept) are commonly used.

Currently, the conventional clinical treatment is based on lifelong progressive drug administration; nevertheless, it is associated with several drawbacks, including high dose and frequency of systemic drug administration, as well as unavoidable systemic adverse effects, such as gastro-intestinal, ocular, hepatic and renal toxicity, infections, cardiovascular risk and anaphylaxis [6,7,8,9,10]. To date, all recommended treatment protocols fail to shut down disease progression, and consequently the main goal of alternative approaches remains to reduce pain, inflammation and hinder further destruction to the joints [11].

Since the poor efficacy of systemic treatments in chronic joint diseases is primarily due to the low drug availability within articular joints, IA administration could offer an interesting therapeutic strategy to increase drug concentration at the site of action, minimizing overall systemic exposure [4,12]. However, the main limitation of delivering drugs directly into joint cavities is their rapid extravasation from the leaky barrier of the inflamed synovia. The half-life of small drug molecules, such as GCs or NSAIDs, which successfully provide symptomatic relief in established RA and RA-like inflammatory arthritis, has been reported to be in the range of 1–2 h [13,14]. Hence, the reduced retention time of therapeutic agents in the targeted tissue requires frequent injections, which are associated with both risk of infection and/or joint disability and unsatisfactory patient compliance [4]. Additionally, repeated IA administrations of GCs could cause permanent damages to the articular cartilage, and systemic adverse events cannot be fully avoided; precautions should be observed in patients with concomitant diseases such as hypertension or diabetes mellitus [5,15].

In the attempt to obtain a long-lasting release of therapeutic agents in the synovial cavity through IA administration, different drug delivery systems have been extensively investigated, such as solid lipid particles, polymeric microparticles (MPs) and nanoparticles (NPs) and their combination (NPs-in-MPs), drug–polymer conjugates, hydrogels, and in situ hydrogels as such or containing conventional MPs or NPs [4,16,17,18,19,20,21,22,23]. Overall, conventional nanoscale drug delivery system-based approaches appear very promising because IA administration appears to be simple and better penetration into cartilage tissue is achieved [4]. NP sizes (40–200 nm), as well as polymer features, such as charge, hydrophilicity and stiffness, should prolong their residence time in synovial fluid and improve cartilage uptake as well as therapeutic efficacy [4,18]. In particular, positively charged NPs were exploited to prolong the retention time in the synovial cavity through electrostatic interactions of positively charge NPs with endogenous hyaluronic acid (negatively charged). Moreover, the electrostatic interactions between NPs and the negatively charged constituents of cartilage facilitate NPs cartilage uptake.

The rationale of this work was to exploit the high expression of Epidermal Growth Factor Receptor (EGFR) in fibroblast-like synoviocytes, which are the cellular component involved in the initiation and perpetuation of destructive joint inflammation [24,25,26,27]. The objective was to formulate an EGFR-targeting nanoparticulate-based delivery system suitable for the prolonged, selective delivery, by IA injection, of drugs inside inflamed synovial fluid. The EGFR-targeting nanoparticulate-based delivery system was designed exploiting the high expression of EGFR in fibroblast-like synoviocytes, which are the primarily cellular component involved in the initiation and perpetuation of destructive joint inflammation [24,25,26,27].

In a previous study [28], EGFR-targeting poly-lactide-*co*-glycolide (PLGA)-based conjugate has been synthetized by covalently linking PLGA, biodegradable polymer approved by FDA for parenteral administration in humans, to GE11 [29,30]. GE11 is a dodecapeptide and it is identified as one of the most efficient EGFR allosteric ligand with no evidence of mitogenic activity and it is able to induce receptor-mediated internalization of liposomes and nanostructures. This new polymeric conjugate, GE11-PLGA, was usefully employed for developing selective EGFR-targeting NPs characterized by *stealth* properties provided by the using of PEGylated PLGA polymer (PEG-PLGA) in the polymeric blending, by enhanced stability in human plasma and good cytocompatibility [31]. GE11-PLGA/PEG-PLGA blend NPs were chosen for preparing EGFR-targeting NPs intended for IA administration. The selection of blending was supported by our preliminary results and the excellent in vivo biocompatibility demonstrated for PEGylated PLA derivatives-based microspheres [32]. GE11-PLGA/PEG-PLGA blend NPs were loaded with DXM, which is the GC representative drug frequently used in the treatment of several inflammatory and autoimmune joint diseases by IA or systemic administration [22,33,34,35,36,37].

DXM loaded GE11-PLGA/PEG-PLGA blend NPs were incorporated into thermosensitive polymer solution which is in the liquid state at room temperature and turns into gel state at physiological temperature, forming a depot formulation in articular joints. The combination of DXM loaded GE11-PLGA/PEG-PLGA blend NPs with thermosensitive polymer solution provides an effective approach to further reduce the leakage of NPs from the synovial fluid. Chitosan (CS) is a natural polymer widely used for preparing in situ thermosensitive hydrogels with peculiar advantages, such as biocompatibility, biodegradability non-toxicity, muco-adhesion and antibacterial properties [38,39,40]. CS and β-glycerophosphate (βGP) were used to developed the composite in situ gelling delivery system (CS/βGP-NPs) by mixing DXM loaded GE11-PLGA/PLGA-PEG blend NPs into the CS-based thermosensitive solution (CS/βG) [41,42,43,44]. Syringeability and cytocompatibility were also evaluated on CS/βGP-NPs formulation.

## 2. Results and Discussion

### 2.1. GE11-PLGA/PEG-PLGA Blend Nanoparticles Loaded with DXM

The efficient loading of DXM inside PLGA-based NPs is a challenging key point [45]. Gomez-Gaete et al. particularly investigated many parameters, such as: (i) the effect of PLGA composition; (ii) the choice of organic solvent and its evaporation rate during NPs formation; (iii) the saturation of the continuous phase; (iv) the addition of a lipid polymer; and (v) the initial mass of DXM respect to polymer mass. The highest drug loading (230 μg/100 mg polymer) was obtained using 100 mg of PLGA (75:25 relative lactide:glycolide composition) in a mixture of acetone-dichloromethane at ratio 1:1 (*v*:*v*) and using 10 mg of initial DXM.

Starting from these considerations and from results published in our previous work [28,31], DXM loaded GE11-PLGA/PEG-PLGA blend NPs were prepared using a 1:10 weight ratio of DXM respect to the polymer matrix which is composed by 1:1 ratio (*w*:*w*) blending of GE11-PLGA and PEG-PLGA. In both copolymers, PLGA was characterized by 75:25 relative lactide:glycolide composition.

To obtain satisfactory DXM encapsulation, DXM loaded GE11-PLGA/PEG-PLGA blend NPs were prepared dissolving DXM in Ethanol (0.7 mL) and mixing it with polymer solution in DMSO followed by NPs preparation. Results reported in Table 1 show that the loading of DXM using Ethanol to DXM solvent was very poor (Batch #DXM2, 280 ± 6 μg of DXM/100 mg of NPs). This finding could be due to the rapid diffusion of Ethanol in the aqueous continuous phase (PVA, 1% *w*/*v*) causing the fast leakage of DXM outside the polymeric matrix during the precipitation phase.

Other solvents were used in combination with DMSO, in particular, CH_2_Cl_2_ (Batch #DXM3) and ethyl acetate (Batch #DXM4). These solvents are poorly miscible with water and they were used to dissolve DXM before adding to the polymeric solution in DMSO. While with CH_2_Cl_2_ the drug loading was not significantly improved (Batch #DXM3, 240 ± 6 μg of DXM/100 mg NPs), with ethyl acetate good drug loading was achieved (Batch #DXM4, 785 ± 1 μg of DXM/100 mg NPs).

The continuous phase saturation with salt was not useful in achieving satisfactory DXM loading (Batch #DXM5, 170 ± 16 μg of DXM/100 mg NPs) confirming data reported in the literature and relative to other polymer/DXM solvent mixture [45].

According to these results, ethyl acetate was selected as well-performing DXM solvent in NPs preparation procedure. It is worth noting that ethyl acetate as well as DMSO may be regarded in *Class III* (ICH Q3C “Impurities: guideline for residual solvents”) as less toxic and lower risk to human health. The use of such safe solvents is highly desirable for eco-friendliness of pharmaceutical manufacturing processes.

As shown in Table 1, no significant modifications of NPs size were highlighted when DXM was added to the NPs formulations, NPs mean size was about 150 nm and consequently suitable for EV injection. Very satisfactory PDIs were recorded (about 1.5) highlighting a narrow size distribution for the NPs batches. All NPs batches were characterized by a negative zeta potential (about −25 mV, Table 1).

**Figure 1:**
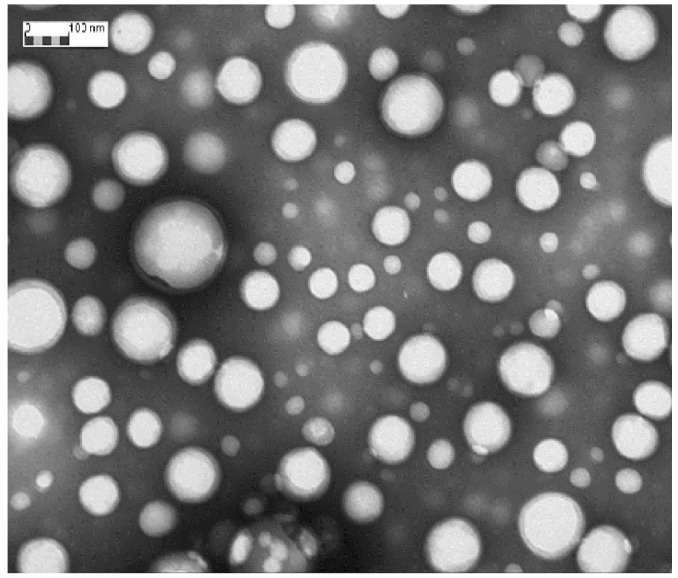
TEM photomicrograph of DXM loaded GE11-PLGA/PEG-PLGA blend NPs (Batch #DXM5). Magnification: 50,000×.

Figure 1 shows TEM image of DXM loaded NPs (Batch #DXM5). DXM loaded GE11-PLGA/PEG-PLGA blend NPs were spherical and regular in shape. No aggregation phenomena have been observed by TEM analysis; data are consistent with the narrow size distribution detected by DLS analysis. TEM images were further elaborated by Jmicrovision software to measure particles size. NPs mean size of 140.40 ± 43.56 nm was calculated confirming the results obtained by DLS analysis.

Process yield of about 80% was estimated for the NPs set-up preparation method.

### 2.2. Nanoparticles Dimensional Stability in Synovial Fluid

Fluorescence Single Particle Tracking (fSPT) is a reliable technique used for evaluating possible aggregations of NPs when they become in contact with biological fluids. In particular, it measures modifications of NPs diffusion coefficient in different biological media compared with the diffusion coefficient in water. These modifications could be due the deposition of protein corona which is responsible of poor stability and fast aggregation of NPs [46].

GE11-PLGA/PEG-PLGA blend NPs are intended to be administered locally by IA route. The final goal of the present research was to treat by in situ treatment different diseases, such as undifferentiated arthritis, limiting the systemic side effects of selected drugs (GCs). The highest efficacy of the nanoparticulate device is guaranteed, unless no aggregation occurs in the site of administration [47]. To this end, the evaluation of NPs stability in synovial fluid becomes the fundamental formulative aspect and fSPT is the only technique available to study the stability of NPs in undiluted biological fluids.

Analyses were performed using fluorescently-labeled NPs formulations [31], both GE11-targeted and untargeted blend NPs prepared using PEG-PLGA previously labeled with PEG-PLGA-RhB have been investigated. The NPs suspension was diluted in synovial fluid withdrawn from patients affected by undifferentiated arthritis; the diluted suspension was stored for 4 h at 37 °C. At specified times (1 and 4 h), NPs sizes were measured through fSPT and compared to NPs sizes at time zero (*t*_0_). Considering the high variability between each collected sample, the viscosity of synovial fluid was measured each time before fSPT experiment with capillary viscometer.

**Figure 2:**
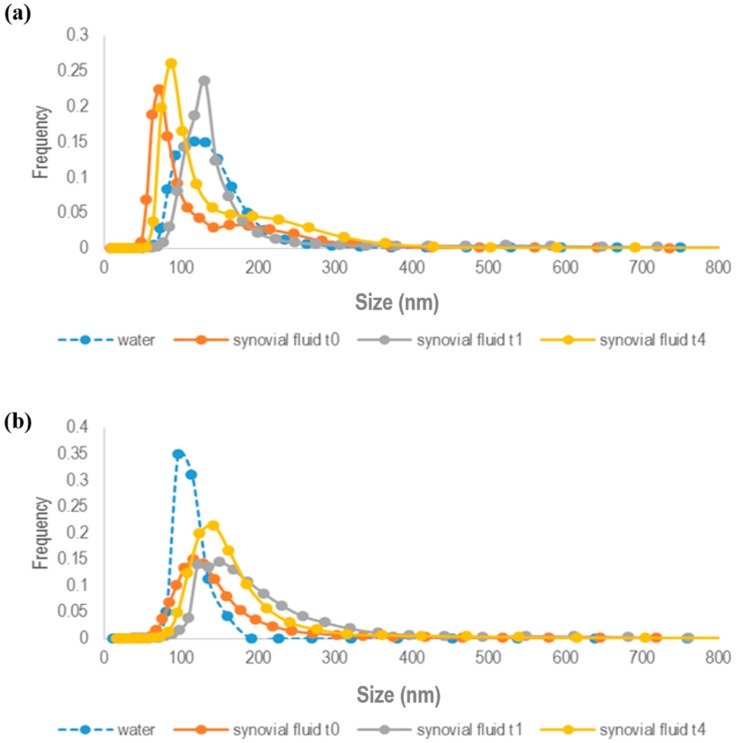
Size distribution of: (**a**) GE11-PLGA/PEG-PLGA-RhB NPs; and (**b**) PLGA/PEG-PLGA-RhB NPs in synovial fluid. Measurements have been carried out by fSPT technique at time zero (*t*_0_), after 1 h (*t*_1_) and 4 h (*t*_4_) of incubation in synovial fluid. The stripped blue line corresponds to NPs size distribution in water and it was used as control.

As shown in Figure 2a,b, both GE11-PLGA/PEG-PLGA-RhB and untargeted PLGA/PEG-PLGA-RhB blend NPs are stable in synovial fluid since no aggregation phenomena were observed. The mean size of untargeted PLGA/PEG-PLGA NPs was increased of about 50 nm after 1 h of incubation in synovial fluid and no further increase in NPs dimension was noticeable at 4 h (Figure 2b).

Since NPs dimension is under 350 nm, it can be stated that NPs are stable and the slight increase in NPs sizes could be attributed to protein absorption (average diameter: *t*_0_ = 130.7 nm; *t*_1_ = 181.9 nm; *t*_4_ = 162.8 nm). The increase in NPs sizes is lower if compared to the increase in dimension due to plasma protein [31]. Indeed, synovial fluid is an ultra-filtrate of blood, so the composition in terms of proteins is the equivalent to the plasma but their concentration is significantly lower compared with the plasma, explaining the reduced adsorption of proteins and the small increase of NPs size.

These findings were consistent with results obtained for GE11-functionalized NPs (Figure 2a) showing a slight increase in NP sizes (average diameter: *t*_0_ = 100.9 nm; *t*_1_ = 145.6 nm; *t*_4_ =130.8 nm) not attributable directly to NPs instability.

In both cases, a slight NPs size increase was revealed when NPs were resuspended in water, even if NPs size was much lower than 350 nm, which is probably due to the major hydrated state of polymeric NPs when in contact with pure water.

The data collected by fSPT technique could be considered as conclusive proof of good stability of GE11 NPs in synovial fluid and their suitable applicability for IA administration.

### 2.3. Cytocompatibility of Nanoparticles

**Figure 3:**
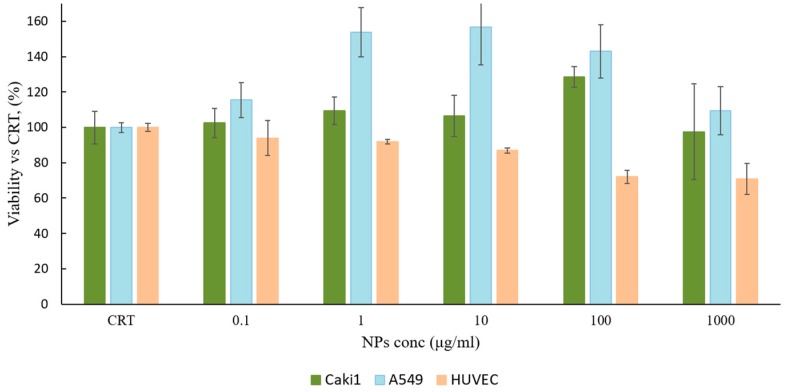
MTS proliferation assay performed on GE11-PLGA/PEG-PLGA blend NPs incubated for 48 h with Caki1, A549 and HUVEC cell lines. The data represent the mean ± sd (*n* = 3, independent experiment). The results are expressed as viability percentage compared to untreated cells (CRT).

PLGA and PEG-PLGA are biodegradable and biocompatible polymers approved by FDA for parental administration and widely used in drug delivery field; however, it is advisable to verify the biological behavior of the new conjugate GE11-PLGA. Cytotoxicity study on placebo GE11-PLGA conjugate-based NPs were carried out on *Caki1* and *A549* cell lines, as overexpressing EGFR cell line models, and HUVEC, as control cell line. Cell viability was evaluated by MTS assay after 48 h of incubation. Results, plotted in Figure 3, were expressed as cell viability percentage (%) compared to the control (CRT, untreated cells).

GE11-PLGA/PEG-PLGA blend NPs highlighted an excellent cytocompatibility. After 48 h of incubation, the cell viability was always higher than 70% for all concentration tested (from 0.1 μg/mL to 1000 μg/mL). Overexpressing EGFR cell line disclosed a good cell viability ranging from 128% to 97% and from 153% to 109% for Caki-1 and A549, respectively. The cell viability decreased until 70% of the control at the highest concentration tested (100–1000 μg/mL) only with HUVEC cell line. As a conclusion, GE11-PLGA/PEG-PLGA blend NPs were found to be safe platform that could be use as drug delivery system in wide range of concentration (0.1–1000 μg/mL).

### 2.4. Incorporation of DXM Loaded GE11-PLGA/PEG-PLGA Blend Nanoparticles into CS-Based Thermosetting Hydrogel

In situ gelling hydrogels have shown great potential in IA drug delivery [21,23]. These hydrogels are easy to handle at room temperature, and they can be readily administered inside the joint because they are characterized by good syringeability and injectability. They undergo sol-gel transition after administration into the body following their exposure to specific stimuli, such as changes in pH or temperature.

The present work envisages the incorporation of targeted GE11-PLGA/PLGA-PEG blend NPs into a CS-based thermosetting hydrogel (CS/βGP). The sol/gel transition of the CS/βGP solution, the gelation mechanism, and their control for potential biomedical application were extensively investigated [43,48,49,50]. The composite hydrogel made of CS/βGP and EGFR-targeted NPs (CS/βGP-NPs) provides a useful delivery platform characterized by distinctive features. CS/βGP could represent an optimal strategy to locally deliver therapeutic agents inside the joints; CS is a biocompatible polymer which can be safely used as delivery device for in situ administration [23,43,44,51]. The gel-like features of CS/βGP would be useful to tune DXM and NPs release from hydrogel formulation inside the joint avoiding the NPs leakage outside the synovial membrane; moreover, the presence of GE11 on the NPs surface could further promote the NPs uptake from EGFR-overexpressing cells improving the selective activity of the drug minimizing systemic and local side effects.

#### 2.4.1. Syringeability of the CS/βGP and CS/βGP-NPs

Syringeability experiments were performed to study the effect of needle on flow rate of CS/βGP and CS/βGP-NPs, at constant pressure of 0.7 bar. Flow rate values higher than 0.4 mL/min are comparable to usual injection rate, as indicated in the literature [52,53]. Results are collected in Table 2.

The experiment showed suitable flow rate value for all sample tested when 20 G needle diameter is used; in addition, no significant differences were highlighted between CS/βGP and CS/βGP-NPs formulations. The flow rate values were 6.63 ± 0.82 mL/min and 6.18 ± 1.03 mL/min for CS/βGP and CS/βGP-NPs, respectively (Table 2). CS/βGP and CS/βGP-NPs results were fully superimposable to reference products for IA administration, namely, hyaluronic acid aqueous solution (HA, 5.42 ± 1.95 mL/min) and GO-ON^®^ (6.17 ± 1.80 mL/min).

All formulations have been further studied in terms of syringeability using syringe-22 G needle system (see Secition 3.2). The results demonstrated that HA was not suitable for syringeability because flow rate was <0.4 mL/min (Table 2), while CS/βGP flow rate (1.73 ± 0.15 mL/min, Table 2) was comparable to the commercial product GO-ON^®^ (1.21 ± 0.05 mL/min, Table 2). Furthermore, reduction in the flow rate was observed for CS/βGP-NPs (0.54 ± 0.07 mL/min) for the 22 G needle system. Both CS/βGP and CS/βGP-NPs formulations can be considered suitable for IA administration through manual injection using slender needles (20 G–22 G) to improve patient compliance.

#### 2.4.2. CS/βGP and CS/βGP-NPs Rheological Characterization

As indicated in the literature, CS/βGP modulates its gelation behavior upon temperature change. This behavior has been ascribed to competition among intermolecular interactions, including electrostatic and hydrogen bonds which implies gradual removal of protective hydration layer formed by the gelling agent around the CS chains. In these conditions, hydrophobic attractive interactions occurred among polymer chains leading to gradual increase of CS/βGP gel strength [41,42,43,48,49,50].

**Figure 4:**
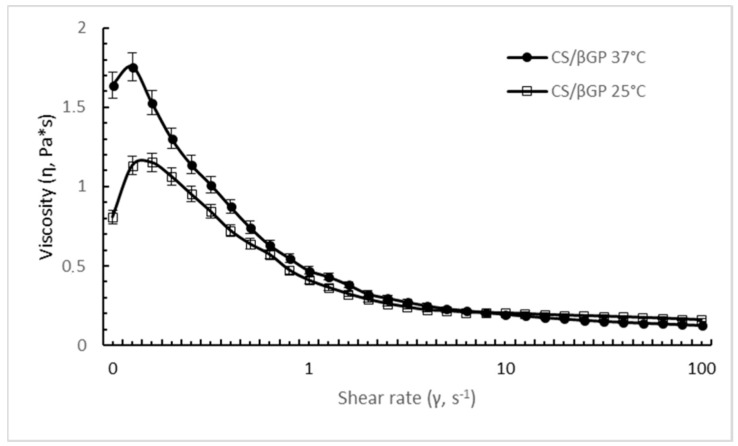
Shear rate viscosity (Pa*s) of thermosensitive CS/βGP formulation as a function of shear rate at 37 °C (body temperature, •) and 25 °C (room temperature, □).

The steady shear behavior of CS/βGP is clearly influenced by the temperature. Figure 4 shows the flow curve of CS/βGP at different temperature; at body temperature (37 °C), a noticeable increase in viscosity was highlighted at low shear rate (<1 s^−1^) compared with formulation measured at room temperature (25 °C). The difference in viscosity was negligible at high shear rate; data could suggest that the increase of temperature enhanced apparent viscosity of CS/βGP triggering the temperature-induced gel structure formation which is broken down by highest shear rates.

The shear-thinning behavior of CS/βGP was still observed at 25 °C and 37 °C; as the shear rate was increased from 0.1 to 100 s^−1^, the viscosity value dropped 7 and 11 folds for the CS/βGP formulation incubated at 25 °C and 37 °C, respectively.

**Figure 5:**
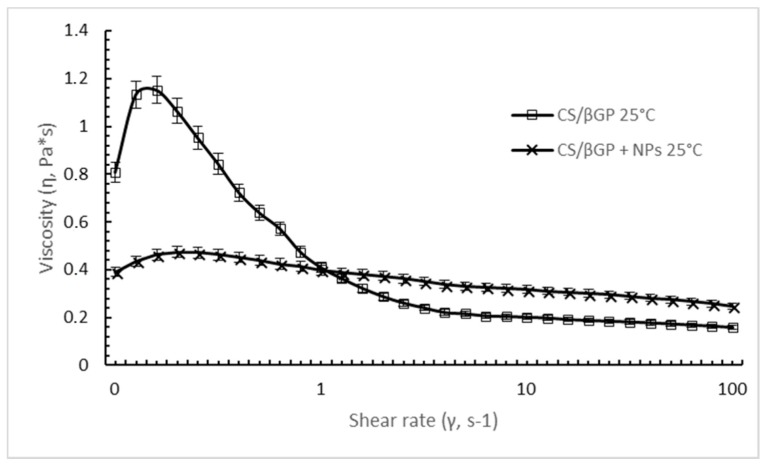
Shear rate viscosity (Pa*s) of thermosensitive CS/βGP (□, empty square) and CS/βGP with DXM loaded PLGA/PEG-PLGA NPs (X, cross) formulation as a function of shear rate applied (0.1–100 s^−1^) at 25 °C.

DXM loaded GE11-PLGA/PEG-PLGA NPs affect the extent of shear thinning behavior of CS/βGP (Figure 5). Although both formulation existing in liquid form at 25 °C, CS solution containing DXM loaded GE11-PLGA/PEG-PLGA NPs showed less remarkable shear-thinning behavior over a broad range of shear rate. Mild downfall of shear viscosity from 0.39 Pa*s to 0.24 Pa*s was detected increasing the shear rate from 0.1 s^−1^ to 100 s^−1^. Moreover, considering the shear rate values applied on polymeric solution during an injection through 20 G–22 G needles (>100 s^−1^), the NPs loaded CS/βGP highlighted a higher shear viscosity (0.24 Pa*s) than CS/βGP alone (0.15 Pa*s) approving the results obtained in the previous syringeability test.

**Figure 6:**
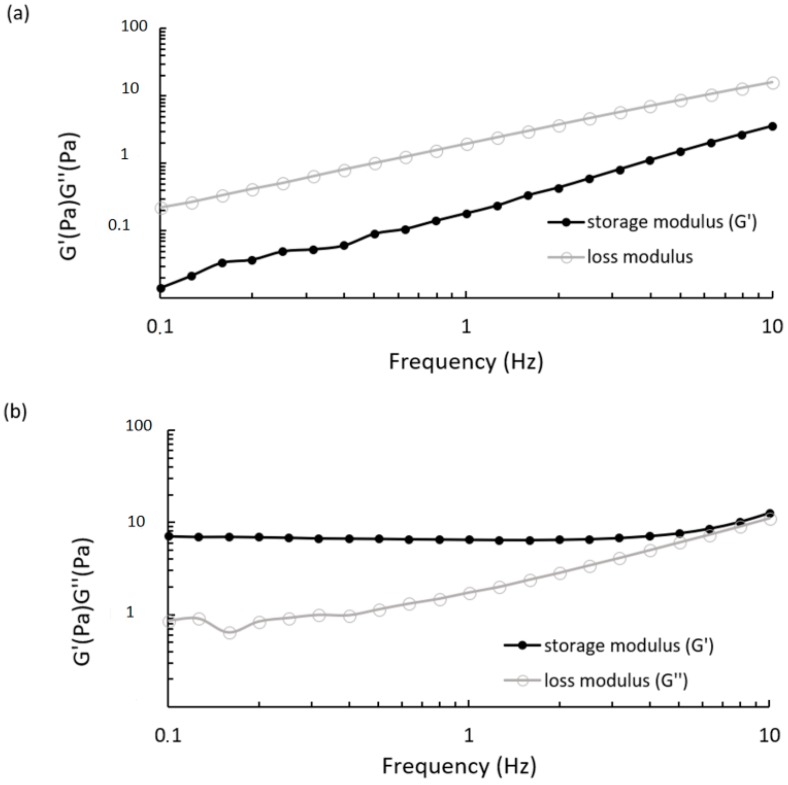
Frequency dependence of storage modulus (G′, •) and loss modulus (G′′, ○) of CS/βGP at: 25 °C (**a**); and 37 °C (**b**). Each modulus was measured between 0.1 and 10 Hz at stress of 0.1 Pa.

Dynamic mechanical analysis provided the viscoelastic properties of CS/βGP at 25 °C and 37 °C measuring the storage modulus (G′) and the loss modulus (G′′) as a function of the oscillatory frequency, 0.1–10 Hz, which is the frequently tested region in rheological studies. As shown in Figure 6a, CS/βGP displayed a loss modulus that was always higher than the loss modulus over the entire frequency range; the most remarkable difference between G and G was observed at lower frequencies (<1 Hz) and both moduli increased with increasing frequencies. These patterns are common for polymeric solution. At 37 °C, oscillatory measurement showed that the storage modulus was higher than the loss modulus over almost the entire frequency range, as reported in Figure 6b. This phenomenon is consistent with the gelation mechanism induced by temperature.

**Figure 7:**
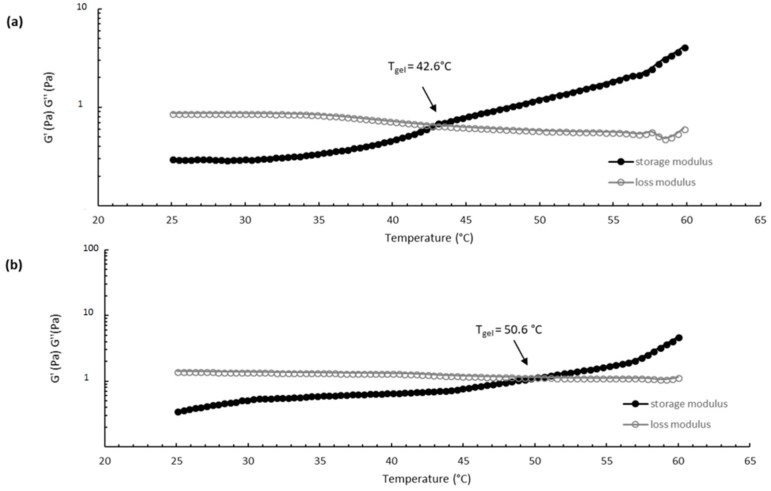
Temperature dependence of storage modulus (G′, •) and loss modulus (G′′, ○) of CS/βGP (**a**) and CS/βGP-NPs (**b**) formulations. The modulus value was measured between 25 and 60 °C (heating rate 5 °C/min, sampling interval = 15 s), at a stress of 0.1 Pa and frequency of 1 Hz.

The thermosensitive properties of CS/βGP and CS/βGP-NPs were evaluated by sol/gel transition temperature determination. The sol/gel transition temperature (T_gel_) corresponds to the temperature at which the storage modulus (G′) is equal to the loss modulus (G′′). CS/βGP exhibits a viscoelastic liquid behavior with the curve showing the dominance of the loss modulus (G′′ > G′) when the temperature is below T_gel_ (T < T_gel_), while the elastic portion became dominant (G′ > G′′), indicating clearly the transition into a gel, as temperature increase at T > T_gel_. The gelation temperature was found to range 40 ± 2 °C and 50 ± 1 °C for CS/βGP and CS/βGP-NPs, respectively (Figure 7). The increased gelation temperature of CS/βGP-NPs formulation was probably due to the presence of negatively charged NPs which could interact electrostatically with positive amine groups of CS polymer preventing polymer chain interactions by steric hindrance during the sol/gel transition.

**Figure 8:**
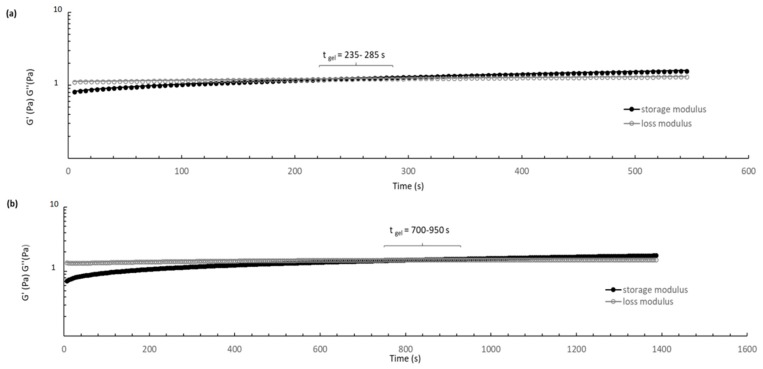
Time dependence of storage modulus (G′, •) and loss modulus (G′′, ○) of CS/βGP (**a**) and CS/βGP-NPs (**b**) formulations. The modulus was measured at 37.5 °C (sampling interval, 5 s) at a stress of 0.1 Pa and frequency of 1Hz.

The suitability of thermosensitive formulation gelation temperature and reproducibility of gel-like behavior in situ was evaluated measuring the sol/gel transition time at 37.5 °C through sweep time test. The tests (Figure 8) showed a considerable increase of the gelation time when DXM loaded PLGA/PLGA-PEG NPs are incorporated in the formulation. CS/βGP and CS/βGP-NPs formulations turned into gel-like state after 5 and 14 min at 37.5 °C, respectively. Gelification times longer than 20 min are not suitable for the intended administration goal, as NPs suspended into the hydrogel may spread out into the surrounding tissue if hydrogel is not quickly formed. Furthermore, once the gelation process began, the moduli gradually increased, indicating the gel network formation is progressive and it was not completed during the measurement time.

No difference in rheological behaviors was highlighted with placebo GE11-PLGA-PEG-PLGA NPs CS thermogelling formulation.

#### 2.4.3. CS/βGP and CS/βGP-NPs Cytocompatibility

Cytocompatibility of thermosetting CS-βGP and CS-βGP containing placebo GE11-PLGA/PEGPLGA blend NPs (CS-βGP-plNPs) was detected by an indirect test as stated by ISO10993-5. The formulations were incubated, after complete gelification in water bath (20 min, at 37 °C), in fresh cell culture medium (1 mL) at 37 °C for seven days. Incubation media toxicity were tested by MTT assay after incubation for 24, 48 and 72 h.

**Figure 9:**
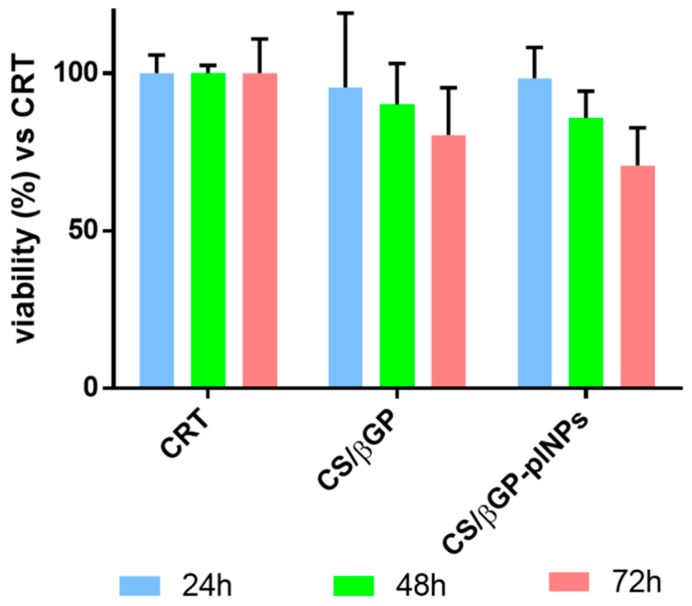
Cytotoxicity of CS-βGP and CS-βGP-plNPs evaluated after incubation for 24, 48 and 72 h with HDFs. Results are presented as mean ± sd (*n* = 3, independent experiment). Tukey′s multiple comparison reveals no significant differences. CRT is untreated cell used and used as control.

Results were expressed as cell viability percentage compared to CRT (untreated cells) as a function of incubation time (Figure 9). Cell viability of CS-βGP and CS-βGP-plNPs formulations was comparable to cells viability of CRT (about 100%) after 24 h of incubation. At Hour 48, CS-βGP and CS-βGP-plNPs formulations presented slight drop of cell viability, ranging from 90% to 86%. After 72 h, cell viability was further reduced; nevertheless, results were higher than 70% compared with CRT. No significant differences were observed between CS-βGP and CS-βGP-plNPs formulations.

These results are the objective evidence of the high biocompatibility of thermosetting CS-βGP which is already widely employed in tissue regeneration for the excellent biological properties of CS. Additionally, the addition of placebo GE11-PLGA/PEG-PLGA NPs inside the gel had no impact on cytocompatibility of composite system CS-βGP-NPs formulation; thus, it can be postulated that selected polymers (GE11-PLGA, PLGA-PEG), solvents and process procedure used for preparing NPs are safe and this assumption is consistent with data previously published [31].

### 2.5. DXM Release

**Figure 10:**
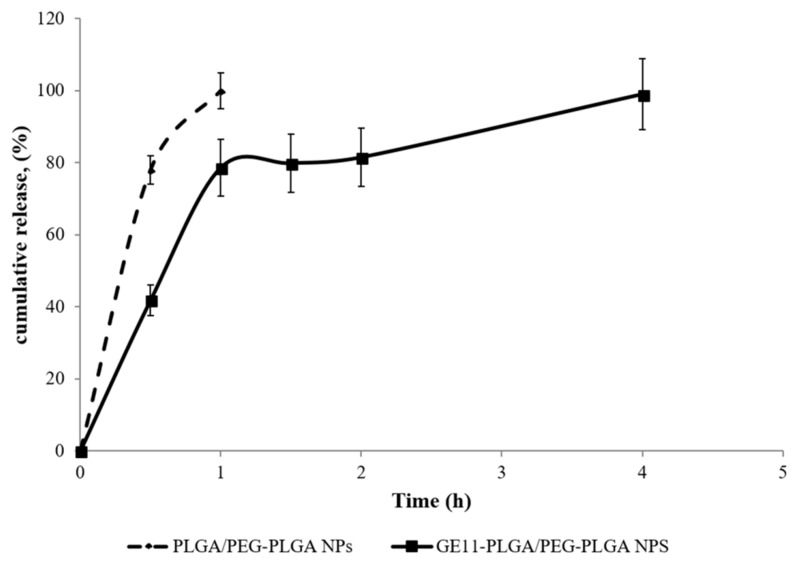
DXM release profile from GE11-PLGA/PEG-PLGA NPs (solid line) and PLGA/PEG-PLGA NPs (dotted line) under simulated inflammatory pathological conditions (pH7.0) at 37 °C.

*In vitro* release test was performed on freeze dried DXM loaded untargeted PLGA/PLGA-PEG NPs and GE11-PLGA/PEG-PLGA NPs suspended in Hepes buffer (2 mL, pH 7.0) at 37 °C, to mimic inflammatory pathological conditions (Figure 10). NPs formulations are characterized by peculiar release profile depending on polymeric composition.

DXM release from PLGA/PEG-PLGA NPs was very rapid; the complete release of the drug was achieved in 60 min, with an initial burst release of about 80% in the first 30 min (Figure 10, dotted line). The high burst release suggests the prevalent drug adsorption on the NPs surface as already observed in previous studies [45].

DXM release profile from GE11-PLGA/PEG-PLGA NPs is composed by three phases (Figure 10, solid line). The first phase is characterized by *burst* release in the first 60 min which consists of about 80% of the DXM loaded amount; in the second phase, the release of remaining drug was completed in 240 min where the plateau is reached. This release pattern could be partially attributed to the amount of DXM not completely loaded in NPs core, to the increased hydrophilicity of polymer matrix containing PEG and GE11, and to the reduced Tg of the polymeric matrix due to the presence of PEG [31]. Nevertheless, the presence of GE11 seems to allow enhanced dispersion of the DXM molecules within the polymer matrix resulting in more prolonged release (within 4 h) compared to untargeted PLGA/PEG-PLGA NPs.

Considering the rapid diffusion of DXM from GE11-PLGA/PEG-PLGA NPs, the studied nano-based formulation cannot represent an advantage as therapeutic device for systemic administration (e.v. injection) compared to the conventional DXM-based solutions or suspensions commonly used in therapy. The almost complete DXM release would be achieved before reaching the target tissue, thus the use of DXM-loaded NPs does not result in the reduction of side effects due to unspecific activity in healthy tissues. On the contrary, the functionalization of PLGA NPs with a targeting agent (GE11), specific for synovial EGFR-overexpressing fibroblasts, could increase DXM efficacy inside the joint following IA administration. Moreover, the NPs selective uptake could enhance the drug efficacy inside the joint. The chance to tune the NPs release rate from the hydrogel, their cellular uptake inside the joints and drug release rate is still challenging.

**Figure 11:**
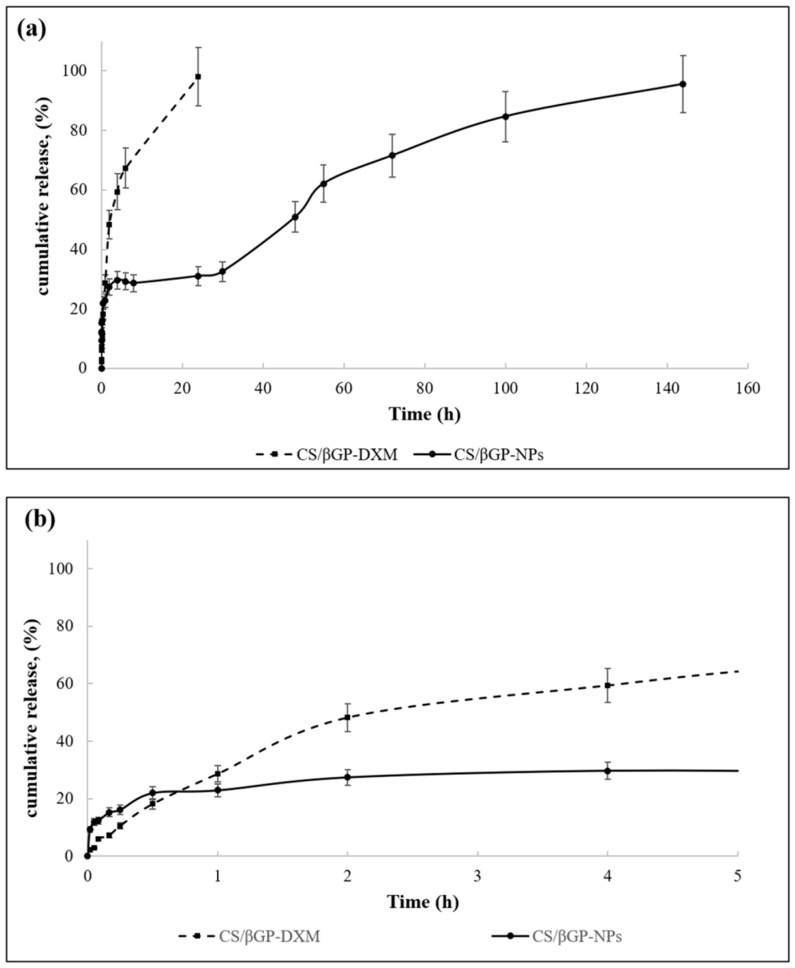
(**a**) DXM release profile from DXM loaded CS/βGP (dotted line) and CS/βGP-NPs (solid line) under simulated inflammatory pathological conditions (pH 7.0) at 37 °C until 140 h of incubation; and (**b**) focus on the first 5 h of incubation.

In this regard, CS/βGP was chosen as potential vehicle for IA injection of targeted NPs. Figure 11 shows DXM release profiles from DXM loaded CS/βGP (CS/βGP-DXM) and the composite formulation CS/βGP-NPs in which DXM loaded GE11-PLGA/PEG-PLGA blend NPs were incorporated into CS/βGP hydrogel. The release test was performed at 37 °C to maintain hydrogel integrity for the full term of test.

The release profile of DXM from CS/βGP is characterized by prolonged release pattern over 24 h (Figure 11a, dotted line,). No *burst* release occurred and about 40% of the loaded drug was slowly released in the first 2 h reaching about 50% at Hour 5 (Figure 11b).

The model that best fits release data was evaluated by correlation coefficients (*R*^2^). The higher correlation coefficient was revealed for Higuchi model, for which a *R*^2^ value of 0.9306 was highlighted. It was demonstrated that the DXM release from the gel matrix is diffusion controlled. Korsmeyer–Peppas model can be used to express drug release from a swellable thin polymeric gel; the diffusional exponent (*n*) was calculated and it was of 0.6523, indicating an anomalous-Fickian behavior [54].

The composite system CS/βGP-NPs showed (black continue line, Figure 11a,b) triphasic release profile (Figure 11a), slightly more rapid release rate could be observed in the first 20 min with respect to CS/βGP-DXM formulation, and the plateau phase was recorded at Hour 5, reaching 20% of release (Figure 11b). DXM release pattern was then characterized by low DXM release rate up to Hour 30 (about 33%, Figure 11a) with following slight increase. CS/βGP-NPs demonstrated the ability to prolong DXM drug retention time within 144 h and to reduce its initial *burst* release.

Considering the rapid release of DXM from NPs and the prolonged DXM release obtained from the composite system CS/βGP-NPs, it is after all realistic to assume that NPs are not quickly released from the hydrogel matrix. Nevertheless, unlike DXM and NPs release profiles could take place when formulation is injected in vivo. When NPs viscous dispersion embedded in a thermosensitive CS-based solution is injected into the joint, NPs potentially have time to partially escape from the CS solution because CS dispersion gelification takes about 15 min at body temperature.

Hence, considering the GE11-PLGA/PLGA-PEG blend NPs’ good size stability in contact with synovial fluid (as previously demonstrated), NPs could be captured by EGFR-overexpressed synovial fibroblasts, decreasing their leakage from the joint and contributing to increase drug efficacy. This speculative hypothesis can be strengthened by the GE11-targeted NPs fast uptake by EGFR-overexpressing cells (≤30 min), as demonstrated by our research group [31] which is compliant with the CS/βGP-NPs gelation time. Subsequently, joints movement can promote gel-structure breakdown allowing further NPs release from the Cs-based hydrogel.

## 3. Materials and Methods

### 3.1. Materials

Poly-(d,l-lactide-*co*-glycolide) (PLGA, 75:25, Mw 30 kDa) and poly-(d,l-lactide-*co*-glycolide)block-poly(ethileneglycole)block-poly(lactide-*co*-glycolide) (PLGA-PEG, 75:25, Mw 36 kDa) were purchased from Lakeshore Biomaterials (Birmingham, AL, USA).

Chitosan chloride (CS) pharmaceutical grade (Protosan CL213, Mw 300–350 kDa, deacetylation degree 82%, hydrochloric acid content 10–20%) was purchased from FMC BioPolymer AS NovaMatrix (Sandvika, Norway); glycerol-2-phosphate disodium salt (β-GP), hyaluronic acid sodium salt (HA), from *Streptococcus equi* (high molecular weight, Mw 1500 kDa) and Dexamethasone (DXM) were from Sigma-Aldrich (Saint Louis, MO, USA). GO-ON^®^ (1% sodium hyaluronate solution, Class III medical device—Xeditone Pharmaceutical Inc., Mississauga, Ontario, Canada) was available on the market. All other reagents, resins and solvents were purchased from Sigma-Aldrich (Saint Louis, MO, USA) and/or Merck KGaA (Darmstadt, Germany) and used without any further purification; all solvents were of HPLC grade.

HUVEC (ATCC^®^ CRL-1730™), Caki-1 (ATCC^®^ HTB-46™), A549 (ATCC^®^ CCL-185™) cells and Normal, Human, Adult, Primary Dermal Fibroblasts (HDFa) (ATCC^®^ PCS-201-012™) were purchased from PromoCell (VWR International PBI s.r.l, Milano, Italy).

Sinovial fluid was recovered from patients affected by undifferentiated arthritis at the Gent University Hospital (Gent, Belgium).

### 3.2. Methods

#### 3.2.1. DXM Loaded GE11-PLGA/PEG-PLGA Blend Nanoparticles Preparation

##### Preparation of Placebo Nanoparticles

GE11 was custom synthetized by Solid Phase Peptide Synthesis according Fmoc methodology using a microwave synthetizer (Biotage Microwave SD Initiator, Uppsala, Sweden) on the basis of a well established method [28,55]. GE11-PLGA conjugate was synthetized using carbodimmide chemistry procedure previously described and set-up [28].

GE11-PLGA conjugate-based NPs were prepared following a modified nanoprecipitation technique suitably set-up for GE11-PLGA/PEG-PLGA blend NPs by our research group [31]. Briefly, NPs were prepared by using a physical mixture of GE11-PLGA and PEG-PLGA co-polymers in a 1:1 weight ratio to obtain GE11-PLGA/PEG-PLGA blend NPs. The physical mixture of polymers (raw materials) was dissolved in 5 mL of DMSO at final concentration of 15 mg/mL. The polymeric solution was then poured into continuous phase (10 mL) containing PVA (1% *w*/*v*), under vigorous stirring. NPs were maintained for 4 h under magnetic stirring (700 rpm) to ensure complete curing. They were washed twice with bi-distilled water in a row and recovered by ultracentrifugation (30 min, 4 °C, 40,000 rpm; Ultracentrifuge Beckman LE80 equipped with a fixed angle rotor Ti70, Beckman Coulter, Pasadena, CA, USA) (Batch #1, Table 1).

Untargeted PLGA/PEG-PLGA blend NPs were prepared using PLGA and PEG-PLGA in the same weight ratio respect to GE11-PLGA conjugate-based NPs. Untargeted NPs were prepared as reference, according to the procedure afore described for GE11-functionalized NPs preparation.

Each NPs batch was prepared at least in triplicate; at the end, after recovery by centrifugation, each NPs batch was suspended in 1 mL of bi-distilled water, frozen at −25 °C overnight and freeze-dried at −50 °C, 0.01 bar for 24 h (Lio 5P, Cinquepascal s.r.l., Milan, Italy). Percentage process yield was calculated as percent ratio between weighted freeze-dried NPs batch and raw materials used in the preparation of each NPs batch.

##### Optimization of DXM Loading into GE11-PLGA Conjugate-Based NPs

The optimization of DXM loading into GE11-PLGA conjugate-based NPs has been performed using different approaches. The first approach was based on using different organic solvents, such as ethanol (EtOH), Methylene Chloride (DCM) or Ethyl Acetate (EAc). In particular, DXM (7.5 mg) was alternatively dissolved in 0.7 mL of each organic solvent (Table 1) before its adding into DMSO polymeric solution. The weight ratio between polymeric mixture and DXM was kept constant at 10:1 *w*:*w* (Batches # DXM2-4, Table 1).

The second approach consisted in increasing the osmotic concentration of the continuous aqueous phase. In particular, Batch #DXM5 (Table 1) was prepared adding sodium chloride (NaCl) to the PVA aqueous solution at final concentration of 5% *w*/*v*.

All the prepared batches are summarized in Table 1. Each batch was prepared at least in triplicate. Process parameters such as polymer concentration, phase volumetric ratio and curing conditions were kept unchanged as set up for placebo batches.

Untargeted PLGA/PEG-PLGA blend NPs loaded with DXM were prepared using the previously selected set-up conditions and used as control. 

For each NPs batch, the percent process yield was calculated as percent ratio between weighted freeze-dried NPs batch and raw materials used in the preparation of each NPs batch.

##### DXM Loading Determination

DXM loading was determined quantifying loaded DXM directly from freeze-dried DXM-loaded NPs powder. Briefly, 5 mg of DXM-loaded NPs were dissolved in DMSO (1 mL) under magnetic stirring. Then, 3 mL of methanol were added to DMSO to ensure polymer precipitation. The obtained suspension was centrifuged (16,400 rpm, 10 min, 4 °C) to separate polymer from DXM. The DXM content in the supernatant was analyzed by HPLC according to an optimized method [45]. The analytical conditions are the following: column C18 Zorbax Eclipse plus, 4.6 × 15 cm, 5 μm; mobile phase, 40:60 (*v*:*v*) Acetonitrile: H_2_O, flow rate 1 mL/min and wavelength 238 nm. The complete run was fixed to 10 min and the DXM peak was detected after 4 min. The calibration curve was prepared dissolving different amounts of DXM in a mixture of DMSO: Methanol at ratio 1:3 (*v*/*v*); good linearity between concentration and absorbance was found between 0.8 and 26 μg/mL of DXM (*y* = 52.31*x* + 1.35, *R*^2^ = 0.99977). The results are expressed as amount of DXM (μg) in 100 mg of NPs.

#### 3.2.2. Characterization of Nanoparticles: Morphology, Size and Surface Charge

NPs shape and morphology were visualized using transmission electron microscopy (TEM 208 S, Philips, Milan, Italy). GE11-PLGA/PEG-PLGA NPs, after centrifugation, were resuspended in water. A small aliquot (15 μL) was stained with 1% (*w*/*v*) phosphotungstenic acid (pH 7.0 adjusted with NaOH solution, 1 M) for 2 min and then it was immobilized on a copper grids to assess the analysis by TEM. TEM micrographs were the elaborated by Jmicrovision 1.0 software (JMicro Visi Geneva, Switzerland) to reveal the NPs size.

Size and size distribution of all prepared NPs batches were further investigated using dynamic light scattering (NICOMP 380 ZLS, Particle Sizing Systems—Entegris, Port Richey, FL, USA). Measurements were performed on NPs resuspended in bi-distilled water. The zeta potential of NPs was evaluated with the same instrument using platinum electrodes; in this case, NPs pellet was resuspended in NaCl aqueous solution, 10 mM. The size analyses and surface charge determinations were performed in triplicate.

#### 3.2.3. Nanoparticles Size stability in Synovial Fluid

NPs dimensional stability in synovial fluid was assessed using Fluorescence Single Particle Tracking (fSPT) [46]. For this purpose, fluorescently-labeled targeted and untargeted fluorescently-labeled NPs were also arranged using Rhodamine B (RhB) as fluorescent marker. Fluorescent NPs were prepared starting from a physical mixture of GE11-PLGA (or PLGA) and PEG-PLGA-RhB conjugate [31], at 1:1 weight ratio, and following the nanoprecipitation method described above. The PLGA-PEG-RhB conjugate was prepared according to carbodiimmide chemistry, as widely described [56].

fSPT experiments were performed on fluorescent NPs samples suspended in synovial fluid collected from patient affected by undifferentiated arthritis at the Gent University Hospital (Gent, Belgium). The collected synovial fluids were characterized in terms of viscosity through capillary viscometer (Capillary viscometer MGW S5, Lauda Brinkmann, Deltran, NJ, USA), and viscosity analyses were carried out before fSPT evaluation.

For the fSPT evaluation, NPs suspensions (10 mg/mL) was diluted 1:300 in PBS. Five microliters of NPs suspension were diluted in 45 μL of synovial fluid and incubated for 1 and 4 h at 37 °C in a glass-bottom 96-well plate (Greiner Bio-One GmbH, Frickenhausen, Germany). During the incubation and measurements, the well plate was sealed with adhesive plates seals (Thermo Scientific, Milan, Italy) to avoid evaporation of sample. At the end of the incubation time, the samples were placed on the custom-built fSPT set-up and movies were recorded at 5 μm above the bottom of the plate. Videos were recorded at 22.5 °C (RT) with the NIS Elements software (Nikon, Tokyo, Japan) driving the CCD camera (Cascade II:512, Roper Scientific, Tucson, AZ, USA) and a TE2000 inverted microscope equipped with a 100_NA1.4 oil immersion lens (Nikon, Tokyo, Japan). Each experiment was performed in triplicate; the results are expressed as dimensional distribution of NPs vs. time of incubation in synovial fluid.

As comparison, analyses were assessed on fluorescently-labelled NPs resuspended in water at equal experimental conditions.

#### 3.2.4. Nanoparticles Cytocompatibility

Cytotoxicity of GE11-PLGA conjugate-based NPs was determined evaluating cells proliferation after incubation with NPs suspension. For this purpose, an MTS test using CellTiter 96^®^ AQueous One Solution kit (Cell Proliferation Assay, Promega, Madison, WI, USA) was performed. The test was performed on Caki1 and A549 cell lines, as overexpressing EGFR cell line models, and HUVEC, as control. Cells were seeded in a 96-well plate, 3000–5000 cells/well. NPs were resuspended in the cell culture media (RPMI, 10% *v*/*v* FBS) at different concentrations (from 0.1 μg/mL to 1 mg/mL); each NPs suspension was added to the cells and further incubated for 48 h at 37 °C, 5% CO_2_.

The results of cell proliferation were expressed as a percentage of vital cells respect to the control, which is characterized by cells not incubated with NPs.

#### 3.2.5. Formulation of Thermosetting CS-βGP and CS-βGP-NPs

CS-βGP was prepared by a combination of chitosan CL213 (CS) and β-glicerophosphate solutions (β-GP) [51]. Briefly, 2.4% *w*/*v* CS solution in bi-distilled water was sterilized for 15 min in autoclave at 121 °C and 1 atm (Autoclave Vapor Matic 770, Milan, Italy). Separately, 58% *w*/*v* β-GP solution was prepared in bi-distilled water and sterilized by filtration using appropriate filter membrane (0.22 μm). Both solutions were cooled down to 4 °C in ice/water bath. β-GP solutions (172.4 µL) were dropped into CS solutions (827.6 µL) in a volume ratio of 1:4.8 and were mixed in an ice bath for about 1 h to allow the hydrogel forming.

CS-βGP-NPs formulation was prepared suspending DXM loaded GE11-PLGA/PLGA-PEG blend NPs (60 mg) into the β-GP solution, thereafter the obtained suspension has been added into chitosan solution, as described before. The amount of NPs (60 mg) was selected considering the DXM loading into NPs and the common DXM dose in prompt-release formulation for IA injection [36].

As control, DXM-loaded CS-βGP was prepared directly dissolving DXM in the β-GP solution; the amount of DXM used was comparable to amount of DXM effectively loaded into 60 mg of NPs.

##### Syringeability of CS-βGP and CS-βGP-NPs

Solution syringeability is the most critical requirement to consider for an IA injection. The syringeability of CS-βGP and CS-βGP-NPs formulations was evaluated using purpose-designed instrumentation, Performus^TM^ III (EFD^®^ Inc., East Providence, RI, USA). The instrument consisted on a 5 cc clear luer lock syringe barrel equipped with white piston; for the analysis, needles (EFD^®^ Inc.) having different gauge were used, namely 20 G (ID 0.61 mm leigh 25.4 mm) and 22 G (ID 0.41 mm leigh 25.4 mm), which are commonly used to improve patient compliance. The syringe was loaded with CS-βGP or GE11-PLGA/PLGA-PEG NPs (2 mL) and all tests were carried out at constant pressure (0.7 bar). The results were reported as flow rate (mL/min); the proper flow rate for manual injection was considered to be higher than 0.4 mL/min [52,53].

The syringeability study was broadened to hyaluronate-based formulations normally employed for IA visco-supplementation [57]: reference Hyaluronic Acid aqueous solution (HA; Mw > 1500 kDa, 1.5% *w*/*v*) and GO-ON^®^ (1% *w*/*v* high molecular weight hyaluronic acid solution in phosphate buffer), the latter an injectable product already available on the market.

##### Rheological Behavior of CS-βGP and CS-βGP-NPs

CS-βGP and CS-βGP-NPs formulations were characterized regarding rheological behavior using a Rheometer Kinexus Plus (Alfatest, Milan, Italy) supplemented with circulated environmental system for temperature control. The sample (2 mL) was carefully placed within cone-plate geometry CP4/40 (1° cone angle, 40 mm diameter) with a gap of about 0.15 mm. The solvent trap was used to minimize solvent evaporation during all analysis.

The temperature flow curve of thermosensitive gels (CS-βGP and CS-βGP-NPs) was determined from 25 °C to 37 °C, while shear rate ramp test runs were assessed increasing shear rate from 0.1 to 100 s^−1^ and viscosity value was measured. Each datum is the mean of triplicate analysis.

Low shear rate behavior can be related to storage stability and levelling, while medium shear rate reflects those properties commonly encountered during spreading and pumping. Rubbing and spraying are examples of high shear processes. The shear rates applied on CS-βGP and CS-βGP-NPs formulations mimic their extrusion through 20–22 G needle (injection phase), using flow rate of >0.4 mL/min, as describe in the literature [58].

Frequency sweep test was performed to measure the viscoelastic response of samples at 25 °C and 37 °C in an oscillation mode (10–0.1 Hz), at constant stress (0.1 Pa). Polymer solution viscoelastic properties were assessed by measuring the storage modulus (G′) and loss modulus (G′′) representing the elastic behavior and viscous behavior of the materials, respectively. Oscillation measurement was carried out in the linear viscoelastic region previously evaluated. Each datum is the mean of at least triplicate analysis.

To evaluate the skill of turning into semisolid hydrogel at body temperature upon injection, temperature sweep test was performed at a constant frequency of 1 Hz and stress 0.1 Pa. The temperature ranged from 25 °C to 60 °C to pinpoint the gelation temperatures. The sol/gel transition temperature (T_gel_) corresponds to the intersection of the storage and loss moduli curves, i.e. when they are equal. The Kinexus Plus rheometer was also used to characterize the time-dependent changes in the elasticity modulus at 37.5 °C, as such process is associated with both the rate and extent of thermosensitive gel curing. Time sweep tests were carried out at constant frequency of 1 Hz at 37.5 °C and gelation time (t_gel_) was identified by the intersection of G′ and G′′ curves.

##### Cytocompatibility of CS-βGP and CS-βGP-NPs

Monolayer human adult dermal fibroblasts (HDFs), purchased as primary cells from International PBI (Milan, Italy), were cultured in DMEM supplemented with 10% *v*/*v* FBS and 1% *v*/*v* antibiotic solution (100 U/mL penicillin and 100 µg/mL streptomycin), in a 96-well plate (10,000 cells/well). Cytocompatibility of thermosetting CS-βGP and CS-βGP containing placebo GE11-PLGA/PLGA-PEG NPs (CS-βGP-plNPs) was detected by an indirect test method as stated by ISO10993-5 [59].

Thermogelling CS solution (1 mL), as such or containing GE11-PLGA/PLGA-PEG NPs (60 mg), was placed in a 24-well plate and maintained at 37 °C in a water bath till to complete gelification (15–20 min). Fresh cell culture medium (1 mL) was gently added to each well and samples incubated at 37 °C for 7 days.

An aliquot of 100 µL of culture medium was withdrawn and seeded on HDFs with 100 µL of fresh DMEM, Samples were incubated at 37 °C, 5% CO_2_ for 24, 48 and 72 h. At the scheduled times, HDFs proliferation was evaluated by an MTT test. All experiments were performed in triplicate

Cytotoxicity of CS-βGP and CS-βGP-NPs was expressed as a percentage of vital cells respect to the control, untreated HDFs, incubated with 200 μL of fresh DMEM.

#### 3.2.6. DXM Release Tests

In vitro release tests were carried out on DXM loaded GE11-PLGA/PEG-PLGA and on DXM loaded PLGA/PEG-PLGA NPs; these results were considered as reference to evaluate hydrogel-like formulations (CS-βGP and CS-βGP-NPs). Briefly, 4.5 mg of NPs were suspended in 2 mL of Hepes buffer (pH 7.0 or 7.4) and gently shaken on a bench-top shaker-incubator (See-saw rocker SSL4, Stuart^®^ equipment, Stafforshire, Stone, UK) at 37 °C. The release test was performed keeping the concentration of DXM under *sink* conditions (DXM solubility in Hepes buffer was 74 μg/mL). At predetermined time points, 1 mL of release medium was withdrawn and replaced with equal volume of fresh Hepes buffer.

DMX release tests from CS-βGP and CS-βGP-NPs were carried out on formulations (1 mL) incubated at 37 °C in a water bath for 15–20 min to promote liquid–gel transition phase. Gel-like formulations were transferred into Hepes buffer (pH 7.0) and the ratio between loaded DXM and the volume of the release medium was adjusted to maintain *sink* conditions (each hydrogelwas soaked into 42 mL of release medium).

Each sample was analyzed by HPLC following the protocol previously described. DXM concentration was determined through a calibration curve between 1.37 and 22 μg/mL of DXM in Hepes buffer (*y* = 49.637*x* + 0.5841; *R*^2^ = 0.9998). Results were expressed as percentage of released DXM respect to loaded DXM in the sample vs. time. All experiments were performed in triplicate.

DXM powder (1.5 mg) was also submitted to a dissolution test in the same experimental conditions described above; 100 mL of Hepes buffer were used to keep *sink* conditions.

#### 3.2.7. Statistical Analysis

Statistical significance was determined by application of two-way analysis of variance (ANOVA) with a Tukey′s multiple comparison test and of multiple t-test (Holm–Sindak method).

Differences were considered significant at *p* value < 0.05. All statistical analyses were performed in GraphPad Prism version 5 (GraphPad Software Inc., La Jolla, CA, USA).

## 4. Conclusions

In the present work, an eco-friendly nanoprecipitation method was set up for the preparation of GE11-PLGA/PEG-PLGA NPs containing high DXM payload. GE11-PLGA conjugate based NPs were characterized by high cytocompatibility, size distribution suitable for IA injection and good size stability in synovial fluid. The presence of GE11 peptide covalently linked to PLGA was assigned to GE11-PLGA/PEG-PLGA NPs selective targeting features towards EGFR-overexpressing fibroblasts involved in chronic joint diseases.

The present work proved that thermogelling composite system CS/βGP-NPs could be a suitable, flexible and promising platform for local treatment of joint diseases. CS/βGP-NPs formulation has been well characterized, including syringeability, rheological and thermo-gelation behavior. The good cytocompatibility of CS/βGP-NPs is the further evidence of CS/βGP-NPs suitability for biomedical applications. These promising results encourage further in vivo studies to identify the most applicable application in the biomedical area.

## Figures and Tables

**Table 1:** Physicochemical properties of GE11-PLGA/PEG-PLGA blend NPs batches: DXM content, size, polydispersity index (PDI) and Zeta potential (Z pot). Results are expressed as mean ± standard deviation (sd).

Batch #	DXM Solvent	Continuous Phase Composition		Drug Content (g DXM/100 mg NPs)	Size ± sd (nm)	PDI ± sd	Z Pot ± sd (mV)
		PVA (%, *w*/*v*)	NaCl (%, *w*/*v*)				
**1**	-	1	-	-	143.9 ± 5.0	0.145 ± 0.030	−24.9 ± 3.4
**DXM2**	Ethanol	1	-	280 ± 6.0	151.3 ± 6.5	0.133 ± 0.028	−26.7 ± 2.6
**DXM3**	CH_2_Cl_2_	1	-	240 ± 6.0	144.4 ± 5.2	0.129 ± 0.029	−21.8 ± 5.5
**DXM4**	Ethyl acetate	1	-	785 ± 1.0	145.7 ± 4.5	0.147 ± 0.032	−23.1 ± 5.3.
**DXM5**	-	1	5	170 ± 16	151.6 ± 2.9	0.126 ± 0.033	−25.9 ± 2.9

Batch #1, placebo NPs, is used as reference.

**Table 2:** Flow rate values of HA (15 mg/mL aqueous solution), GO-ON^®^, CS/βGP and CS/βGP-NPs using a syringe-needle system with 22 G and 20 G needle. Results are reported as mean ± sd (*n* = 3 independent experiments).

Sample	Flow Rate Mean Value ± sd (mL/min)	
	22 G	20 G
HA	0.30 ± 0.01	5.42 ± 1.95
GO-ON^®^	1.21 ± 0.05	6.17 ± 1.80
CS/βGP	1.73 ± 0.15	6.63 ± 0.82
CS/βGP-NPs	0.54 ± 0.07	6.18 ± 1.03

## References

[1] Larsen C., Ostergaard J., Larsen S.W., Jensen H., Jacobsen S., Lindegaard C., Andersen P.H. (2008). Intra-articular depot formulation principles: Role in the management of postoperative pain and arthritic disorders. J. Pharm. Sci..

[2] Helmick C.G., Felson D.T., Lawrence R.C., Gabriel S., Hirsch R., Kwoh C.K., Liang M.H., Kremers H.M., Mayes M.D., Merkel P.A. (2008). Estimates of the Prevalence of Arthritis and Other Rheumatic Conditions in the United States Part I. Arthritis Rheum..

[3] Tonge D.P., Pearson M.J., Jones S.W. (2014). The hallmarks of osteoarthritis and the potential to develop personalised disease-modifying pharmacological therapeutics. Osteoarthritis Cartilage.

[4] Ho M.J., Kim S.R., Choi Y.W., Kang M.J. (2018). Recent advances in intra-articular drug delivery systems to extend drug retention in joint. J. Pharm. Investig..

[5] Yang M., Feng X., Ding J., Chang F., Chen X. (2017). Nanotherapeutics relieve rheumatoid arthritis. J. Control. Release.

[6] Scarpignato C., Hunt R.H. (2010). Nonsteroidal anti-inflammatory drug-related injury to the gastrointestinal tract: Clinical picture, pathogenesis, and prevention. Gastroenterol. Clin. N. Am..

[7] Peponis V., Kyttaris V.C., Chalkiadakis S.E., Bonovas S., Sitaras N.M. (2010). Ocular side effects of anti-rheumatic medications: What a rheumatologist should know. Lupus.

[8] Khan Z.A., Tripathi R., Mishra B. (2012). Metotrexate: A detailed review on drug delivery and clinical aspects. Expert Opin. Drug Deliv..

[9] Howard P.A., Delafontaine P. (2004). Nonsteroidal anti-inflammatory drugs and cardiovascular risk. J. Am. Coll. Cardiol..

[10] Singh J.A., Cameron C., Noorbaloochi S., Cullis T., Tucker M., Christensen R., Ghogomu E.T., Coyle D., Clifford T., Tugwell P. (2015). Risk of serious infection in biological treatment of patients with rheumatoid arthritis: A systematic review and meta-analysis. Lancet.

[11] Zhang W., Moskowitz R.W., Nuki G., Abramsom S., Altman R.D., Arden N., Bierma-Zeinstra S., Brandt K.D., Croft P., Doherty M. (2008). OARSI recommendations for the management of hip and knee osteoarthritis, Part II: OARSI evidence-based, expert consensus guidelines. Osteoarthr. Cartilage.

[12] Niguyen C., Lefevre-Colau M.M., Poiraudeau S., Rannou F. (2016). Evidence and recommendations for use of intra-articular injections for knee osteoarthritis. Ann. Phys. Rehabil. Med..

[13] Owen S.G., Francis H.W., Roberts M.S. (1994). Disappearance kinetics of solutes from synovial fluid after intra-articular injection. Br. J. Clin. Pharmacol..

[14] Park C.W., Ma K.W., Jang S.W., Son M., Kang M.J. (2014). Comparison of piroxicam pharmacokinetics and anti-inflammatory effect in rats after intra-articular and intramuscular administration. Biomol. Ther..

[15] Rau R. (2014). Glucocorticoid treatment in rheumatoid arthritis. Expert Opin. Pharmacother..

[16] Thakkar H., Kumar Sharma R., Murthy R.S. (2007). Enhanced retention of celocoxib-loaded solid lipid nanoparticles after intra-articular administration. Drugs R D.

[17] Sandker M.J., Duque L.F., Redout E.M., Chan A., Que I., Löwik C.W.G.M., Klijnstra E.C., Kops N., Steendam R., van Weeren R. (2017). Degradation, intra-articular retention and biocompatibility of monospheres composed of [PDLLA-PEG-PDLLA]-b-PLLA multi-block copolymers. Acta Biomater..

[18] Pradal J., Maudens P., Gabayb C., Seemayer C.A., Jordan O., Allémann E. (2016). Effect of particle size on the biodistribution of nano- and microparticles following intra-articular injection in mice. Int. J. Pharm..

[19] Chen Z., Liu D., Wang J., Wu L., Li W., Chen J., Cai B.-C., Cheng H. (2014). Development of nanoparticles-in-microparticles system for improved local retention after intra-articular injection. Drug Deliv..

[20] Wang D., Miller S.C., Liu X.-M., Anderson B., Wang X.S., Goldring S.R. (2007). Novel dexamethasone-HPMA copolymer conjugate and its potential application in treatment of rheumatoid arthritis. Arthrit. Res. Ther..

[21] He Z., Wang B., Hu C., Zhao J. (2017). An overview of hydrogel-based intra-articular drug delivery for the treatment of osteoarthritis. Colloids Surf. B Biointerfaces.

[22] Son A.R., Kim D.Y., Park S.H., Jang J.Y., Kim K., Kim B.J., Yin X.Y., Kim J.H., Min B.H., Han D.K. (2015). Direct chemotherapeutic dual drug delivery through intra-articular injection for synergistic enhancement of rheumatoid arthritis treatment. Sci. Rep..

[23] Qi X., Qin X., Yang R., Qin J., Li W., Luan K. (2016). Intra-articular administration of chitosan thermosensitive in situ hydrogels combined with diclofenac sodium-loaded alginate microspheres. J. Pharm. Sci..

[24] Swanson C.D., Akama-Garren E.H., Stein E.A., Petralia J.D., Ruiz P.J., Edalati A., Lindstrom T.M., Robinson W.H. (2012). Inhibition of Epidermal Growth Factor Receptor Tyrosine kinase ameliorates collagen-induced arthritis. J. Immunol..

[25] Yuan F.-L., Li X., Lu W.-G., Sun J.-M., Jiang D.-L., Xu R.-S. (2013). Epidermal growth factor receptor (EGFR) as a therapeutic target in rheumatoid arthritis. Clin. Rheumatol..

[26] Niu J., Li C., Jin Y., Xing R., Sunb L., Yu R., Jian L., Liu X., Yang L. (2018). Identification and suppression of epidermal growth factor receptor variant III signaling in fibroblast-like synoviocytes from aggressive rheumatoid arthritis by the mimotope. Immunol. Lett..

[27] Huang C.-M., Chen H.-H., Chen D.-C., Huang Y.-C., Liu S.-P., Lin Y.-J., Chang Y.-Y., Lin H.-W., Chen S.-Y., Tsai F.-J. (2017). Rheumatoid arthritis is associated with rs17337023 polymorphism and increased serum level of the EGFR protein. PLoS ONE.

[28] Colzani B., Biagiotti M., Speranza G., Dorati R., Modena T., Conti B., Tomasi C., Genta I. (2014). Smart biodegradable nanoparticulate materials: Poly-lactide-*co*-glycolide functionalization with selected peptides. Curr. Nanosci..

[29] Li Z., Zhao R., Wu X., Sun Y., Yao M., Li J., Xu Y., Gu Y. (2005). Identification and characterization of a novel peptide ligand of epidermal growth factor receptor for targeted delivery of therapeutics. FASEB J..

[30] Genta I., Chiesa E., Colzani B., Modena T., Conti B., Dorati R. (2018). GE11 peptide as an active targeting agent in antitumor therapy: A minireview. Pharmaceutics.

[31] Colzani B., Speranza G., Dorati R., Conti B., Modena T., Bruni G., Zagato E., Vermeulen L., Dakwar G.R., Braeckmans K. (2016). Design of smart GE11-PLGA/PEG-PLGA blend nanoparticulate platforms for parenteral administration of hydrophilic macromolecular drugs: Synthesis, preparation and in vitro/ex vivo characterization. Int. J. Pharm..

[32] Te Boekhorst B.C.M., Jensen L.B., Colombo S., Varkouhi A.K., Schiffelers R.M., Lammers T., Storm G., Nielsen H.M., Strijkers G.J., Foged C. (2012). MRI-assessed therapeutic effects of locally administered PLGA nanoparticles loaded with anti-inflammatory siRNA in a murine arthritis model. J. Control. Release.

[33] Butoescu N., Jordan O., Burdet P., Stadelmann P., Petri-Fink A., Hofmann H., Doelker E. (2009). Dexamethasone-containing biodegradable superparamagnetic microparticles for intra-articular administration: Physicochemical and magnetic properties, in vitro and in vivo drug release. Eur. J. Pharm. Biopharm..

[34] Park J.S., Yang H.N., Jeon S.Y., Woo D.G., Kim M.S., Park K.-H. (2012). The use of anti-COX2 siRNA coated onto PLGA nanoparticles loading dexamethasone in the treatment of rheumatoid arthritis. Biomaterials.

[35] Grodzinsky A.J., Wang Y., Kakar S., Vrahas M.S., Evans C.H. (2017). Intra-articular dexamethasone to inhibit the development of post-traumatic osteoarthritis. J. Orthop. Res..

[36] Matzkin E.G., Curry E.J., Kong Q., Roger M.J., Henry M., Smith E.L. (2017). Efficacy and treatment response of intra-articular corticosteroid injections in patients with symptomatic knee osteoarthritis. JAAOS.

[37] Zhang Z., Wei X., Gao J., Zhao Y., Zhao Y., Guo L., Chen C., Duan Z., Li P., Wei I. (2016). Intra-Articular Injection of Cross-Linked Hyaluronic Acid-Dexamethasone Hydrogel Attenuates Osteoarthritis: An Experimental Study in a Rat Model of Osteoarthritis. Int. J. Mol. Sci..

[38] Baldrick P. (2010). The safety of chitosan as a pharmaceutical excipient. Regul. Toxicol. Pharmcol..

[39] Yin H., Du Y., Zhang J. (2009). Low molecular weight and oligomeric chitosans and their bioactivities. Curr. Top. Med. Chem..

[40] Supper S., Anton N., Boisclair J., Seidel N., Riemenschnitter M., Curdy C., Vandamme T. (2014). Chitosan/glucose 1-phosphate as new stable in situ forming depot system for controlled drug delivery. Eur. J. Pharm. Biopharm..

[41] Chenite A., Chaput C., Wang D., Combes C., Buschmann M.D., Hoemann C.D., Leroux J.C., Atkinson B.I., Binette F., Selmani A. (2000). Novel injectable neutral solutions of chitosan form biodegradable gels in situ. Biomaterials.

[42] Cho J., Heuzey M.C., Bégin A., Carreau P.J. (2005). Physical gelation of chitosan in the presence of beta-glycerophosphate: The effect of temperature. Biomacromolecules.

[43] Dorati R., Colonna C., Genta I., De Trizio A., Modena T., Kloss H., Conti B. (2015). In vitro characterization of an injectable in situ forming composite system for bone reconstruction. Polym. Degrad. Stabil..

[44] Genta I., Colonna C., Conti B., Caliceti P., Salmaso S., Speziale P., Pietrocola G., Chiesa E., Modena T., Dorati R. (2016). CNA-loaded PLGA nanoparticles improve humoral response against *S. aureus*-mediated infections in a mouse model: Subcutaneous *vs* nasal administration strategy. J. Microencapsul..

[45] Gómez-Gaete C., Tsapis N., Besnard M., Bochot A., Fattal E. (2007). Encapsulation of dexamethasone into biodegradable polymeric nanoparticles. Int. J. Pharm..

[46] Braeckmans K., Buyens K., Bouquet W., Vervaet C., Joye P., De Vos F., Plawinski L., Doeuvre L., Angles-Cano E., Sanders N.N. (2010). Sizing nanomatter in biological fluids by fluorescence single particle tracking. Nano Lett..

[47] Gaumet M., Vargas A., Gurny R., Delie F. (2008). Nanoparticles for drug delivery: The need for precision in reporting particle size parameters. Eur. J. Pharm. Biopharm..

[48] Chenite A., Buschmann M., Wang D., Chaput C., Kandani N. (2001). Rheological characterisation of thermogelling chitosan/glycerol-phosphate solutions. Carbohydr. Polym..

[49] Filion D., Lavertu M., Bushmann M.D. (2007). Ionization and solubility of chitosan solutions related to thermosensitive chitosan/glycerol-phospate systems. Biomacromolecules.

[50] Lavertu M., Bushmann M.D., Filion D. (2008). Heat-induced transfer of protons from chitosan to glycerol phosphate produces chitosan precipitation and gelation. Biomacromolecules.

[51] Dorati R., De Trizio A., Genta I., Merelli A., Modena T., Conti B. (2016). Formulation and in vitro characterization of a composite biodegradable scaffold as antibiotic delivery system and regenerative device for bone. J. Drug Deliv. Sci. Technol..

[52] Burckbuchler V., Mekhloufi G., Giteau A.P., Grossiord J.L., Huille S., Agnely F. (2010). Rheological and syringeability properties of highly concentrated human polyclonal immunoglobulin solutions. Eur. J. Pharm. Biopharm..

[53] Cilurzo F., Selmin F., Minghetti P., Adami M., Bertoni E., Lauria S., Montanari L. (2011). Injectability evaluation: An open issue. AAPS PharmSciTech.

[54] Ritger P.L., Peppas N.A. (1987). A simple equation for description of solute release II. Fickian and anomalous release from swellable devices. J. Control. Release.

[55] Amblard M., Fehrentz J.A., Martinez J., Subra G. (2006). Methods and Protocols of modern solid phase peptide synthesis. Mol. Biotechnol..

[56] Costantino L., Gandolfi F., Tosi G., Rivasi F., Vandelli M.A., Forni F. (2005). Peptide-derivatized biodegradable nanoparticles able to cross the blood-brain barrier. J. Control. Release.

[57] Cooper C., Rannou F., Richette P., Bruyere O., Al-Daghri N., Altman R.D., Brandi M.L., Collaud Basset S., Herrero-Beaumont G., Migliore A. (2017). Use of Intraarticular Hyaluronic Acid in the Management of Knee Osteoarthritis in Clinical Practice. Arthrit. Care Res..

[58] Kolbuk D., Guimond-Lischer S., Sajkiewicz P., Maniura-Weber K., Fortunato G. (2015). The effect of selected Electrospinning parameters on molecular structure of Polycaprolactone nanofibers. Int. J. Polym. Mater. Polym. Biomater..

[59] Li W., Zhou J., Xu Y. (2015). Study of the in vitro cytotoxicity testing of medical devices (Review). Biomed. Rep..

